# Preparation of Eu(III) Luminescent Hybrid Nanomaterials via Oxidation Induced by Gas-Phase Vacuum Evaporation Approach and Their Anti-Counterfeiting Applications

**DOI:** 10.3390/nano16120741

**Published:** 2026-06-13

**Authors:** Wenzhe Wu, Shaofeng Chen, Wei Ling, Yiwei Tang, Yuji Du, Peilin Liang, Shi-Jian Su, Dongcheng Chen

**Affiliations:** 1 State Key Laboratory of Luminescent Materials and Devices, South China University of Technology, Guangzhou 510640, China; 2 Institute of Polymer Optoelectronic Materials and Devices, South China University of Technology, Guangzhou 510640, China

**Keywords:** divalent europium ions, trivalent europium ions, gas-phase vacuum evaporation approach, anti-counterfeiting

## Abstract

Europium (Eu) is a rare-earth element with unique optoelectronic properties that underpin its applications in displays and lighting, X-ray imaging, anti-counterfeiting, and biomedicine. Conventional methods typically involve the synthesis of europium-based luminescent materials in powder or crystalline form via high-temperature solid-state reactions or solution processes, followed by secondary processing such as spin coating or evaporation to fabricate films or devices. In this work, we report a direct approach to prepare trivalent europium-based luminescent materials using divalent europium bromide (EuBr_2_) as the precursor via a gas-phase vacuum evaporation approach (GPVEA). This “deposition-as-synthesis” method enables the fabrication of the hybrid nanoscale films with various blending ratios, which exhibit changes in the fine structure of the emission peaks. The luminescence spectra remain nearly identical across the temperature range from 80 K to 320 K. The photoluminescence emission intensity is stronger in air than in a vacuum. The films show a maximum photoluminescence quantum yield (PLQY) of 8.27% and good photostability, with an emission decay of 3.44% over 50 min under continuous 300 nm excitation. Through patterned design, we demonstrate their value for anti-counterfeiting applications. This work thus provides guidance for the preparation of europium-based luminescent nanomaterials via GPVEA and their application in anti-counterfeiting.

## 1. Introduction

Europium (Eu) is a rare-earth element with a unique electron configuration, which imparts its characteristic luminescence. This property enables important applications in displays and lighting [1,2], X-ray imaging [3], anti-counterfeiting [4,5,6,7], and biomedicine [8,9,10]. Europium ion typically exists in two oxidation states, divalent [Eu(II)] and trivalent [Eu(III)], which exhibit markedly distinct luminescence properties. The luminescence of divalent europium ions arises from the electronic transition from the 4f^6^5d^1^ excited state to the 4f^7^ ground state, producing broad-band emission covering the range from ultraviolet to red [11,12,13,14]. As this transition involves outer-shell 5d electrons, the corresponding energy levels are strongly influenced by the crystal field and coordination environment, resulting in high tunability of the emission wavelength [15]. In contrast, the 4f orbitals of trivalent europium ions are effectively shielded by the outer closed 5s^2^5p^6^ shell [16]. Their luminescence arises from intra-configurational 4f-4f electronic transitions, giving rise to a series of sharp characteristic emission peaks [1,4,17,18,19]. The luminescence-related energy levels are barely affected by the ligand or solvent environment, thus delivering exceptionally high color purity and stability [20].

Existing preparation processes for europium-based luminescent materials can be categorized into two main classes based on the reaction phase: solid-phase methods and liquid-phase methods. Represented by the high-temperature solid-state reaction route, solid-phase approaches achieve the doping of europium ions into host matrices such as phosphates and borates via high-temperature calcination, and are widely adopted techniques for the industrial production of phosphors [12,21,22]. Liquid-phase methods mainly include routes such as co-precipitation [23], sol–gel processing [11], and hydrothermal synthesis [24,25], where precursors are prepared through uniform mixing of ions in solution systems. However, the solid-state calcination method requires very high temperatures, and the solution-based method requires a solvent during the reaction and subsequent purification of the product. Both methods require the prior synthesis of powder or crystalline materials with specific luminescence properties, followed by secondary processing such as spin-coating and vacuum evaporation to fabricate devices adapted to specific application scenarios [1,2], a step that imposes additional requirements on the material properties, including thermodynamic stability and solubility.

Vacuum evaporation, a type of vapor deposition technology, has been widely used for the fabrication of thin films and devices [26], most notably in the organic light-emitting diodes (OLEDs) display industry [27,28]. Thin films prepared via this method exhibit high uniformity, enable high pattern resolution, and the process itself offers excellent controllability. Another vapor-phase technique, atomic layer deposition (ALD), enables precise thickness control at the atomic scale but suffers from low deposition rates, limited precursor availability, and a narrow temperature window for stable self-limiting growth [29,30]. These drawbacks make ALD inefficient for rapid thick-film deposition and less compatible with high-throughput production lines. In contrast, the development of novel europium-based luminescent materials based on vacuum evaporation technology is expected to achieve direct compatibility with existing industrial production lines, thereby reducing the cost of technological iteration. In addition, most europium-based luminescent materials exhibit phenomena such as emission spectrum shifts under varying temperatures [20,31,32], which limit their anti-counterfeiting applications under extreme conditions, such as low temperatures.

In this work, we employed a “deposition-as-synthesis” strategy using a gas-phase vacuum evaporation approach (GPVEA) to co-evaporate 4,7-diphenyl-1,10-phenanthroline (Bphen) and europium(II) bromide EuBr_2_ from two sources, thereby preparing an organic/inorganic hybrid nanofilm that exhibits trivalent europium ion luminescence. Unlike ALD, GPVEA does not rely on sequential precursor pulses, volatile metal–organic precursors, or a critical temperature window; instead, it uses simple physical co-evaporation, enabling high deposition rates and direct compatibility with existing vacuum production lines, like OLEDs. By tuning the blending ratio of Bphen to EuBr_2_, the emission spectra of the blend films exhibited a composition-dependent evolution of peak positions. Low-temperature photoluminescence spectroscopy measurements revealed that the blend films maintained high consistency of luminescence spectra over a wide temperature range. Furthermore, the films showed higher luminescence intensity in air than in a vacuum. Kinetic measurements under ambient air confirmed the excellent air stability of the films. These results demonstrate that the as-prepared blend films combine tunable optical properties with outstanding environmental adaptability, making them promising for optical anti-counterfeiting. Due to the controllable deposition of nanofilms, we anticipated their applications in optoelectronics. This work offers a new route to fabricate rare-earth functional materials via GPVEA.

## 2. Materials and Methods

Both EuBr_2_ (3AMaterials^®^, Shanghai, China) and Bphen (Changchun Friendcar Technology Co., Ltd., Changchun, China) were deposited onto 1.5 cm × 1.5 cm quartz substrates (Lianyungang Ningtai Quartz Products Co., Ltd., Lianyungang, China). The substrates were placed on a holder inside the vacuum chamber and rotated at 25 rpm. Thin films were deposited under a vacuum of less than 2 × 10^−3^ Pa by resistive heating of a source, and the vertical distance between the evaporation source and the substrate is specified as 35 cm. The deposition rate was monitored using a quartz crystal microbalance (SQC-310, INFICON, NY, USA), which measures changes in the oscillation frequency of the quartz crystal. Up to four different deposition conditions were set per pump-down cycle. To systematically investigate the properties of films with different blending ratios, all samples with various blending ratios were prepared in a single batch, with each deposition condition corresponding to one sample. Patterned deposition was achieved by attaching a patterned shadow mask onto the quartz substrate.

Steady-state photoluminescence spectra were measured with a fluorescence spectrometer (HiLight 990, Oriental Spectra, Guangzhou, China) coupled to a nitrogen cryostat (Optistat DN, Oxford Instrument, Abingdon, UK), using a xenon lamp as the excitation source. Photoluminescence quantum yield was measured using an integrating sphere coupled to the same spectrometer system. Time-resolved photoluminescence decay spectra were acquired with the same spectrometer. For nanosecond- and microsecond-scale excited-state lifetimes, a 307 nm nanosecond diode laser served as the excitation source, whereas a microsecond flashlamp was used for millisecond-scale lifetimes. Raman spectra were recorded using a confocal laser micro-Raman spectrometer (InVia, Renishaw, Wotton-under-Edge, UK) with a 532 nm excitation laser. Unless otherwise noted, all measurements were performed in air at 298 K.

## 3. Results and Discussion

**Figure 1:**
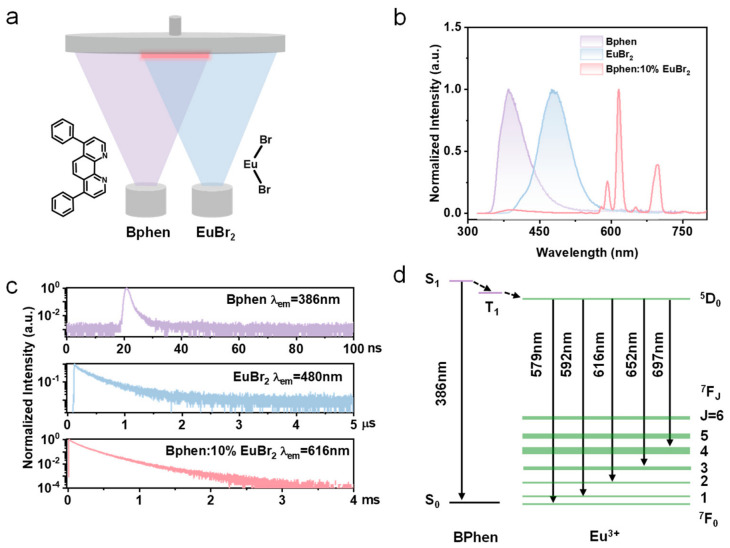
(**a**) Schematic illustration of the gas-phase vacuum evaporation approach; (**b**) photoluminescence spectra; (**c**) time-resolved fluorescence decay curves of Bphen, EuBr_2_, and Bphen: 10% EuBr_2_ blended films; (**d**) the energy level diagram of Eu(III) ions and Bphen, together with the energy transfer between them.

Bphen and EuBr_2_ were co-deposited by heating separately in two independent sources, as shown in Figure 1a. The blending ratio was defined as the ratio of the deposition thickness of EuBr_2_ to the total deposition thickness. Figure 1b shows the photoluminescence (PL) spectrum of a blend film with a EuBr_2_ mixing ratio of 10% (denoted as Bphen:10% EuBr_2_) under excitation at 300 nm. For comparison, the PL spectra of single-component Bphen and EuBr_2_ films are also presented. The Bphen film exhibited an emission peak centred at 386 nm under photoexcitation, whereas the pure EuBr_2_ film showed a blue emission peak at 480 nm. Interestingly, apart from a weak emission from the Bphen ligand at 386 nm, the PL spectrum of the blend film displayed characteristic emission peaks at 579, 592, 616, 652 and 697 nm. To further elucidate the origin of the emission peaks, the excited-state lifetimes at the corresponding wavelengths of each emission peak were measured, and the results are shown in Figure 1c. The excited-state lifetime of the Bphen film at 386 nm was 0.480 ns, that of the EuBr_2_ film at 480 nm was 0.765 μs, and that of the blend film at 616 nm was 0.265 ms. The order-of-magnitude differences in these lifetimes indicate that the emission in the blend film originates from different energy levels of Bphen and Eu(II) ions. The emission at 386 nm is attributed to the fluorescence from the singlet state of Bphen. The sharp characteristic emission peaks in the hybrid film are attributed to electronic transitions from the ^5^D_0_ excited state to the ^7^F_J_ of trivalent Eu(III) ions [10,33], as illustrated in Figure 1d. This finding confirms that the valence state of europium ions in the as-prepared blend film changes from Eu(II) to Eu(III). Under photoexcitation, the organic ligand in the blended film is excited to its singlet state. Energy is then transferred from the singlet to the triplet state of the ligand, followed by further transfer to Eu(III) ions.

**Figure 2:**
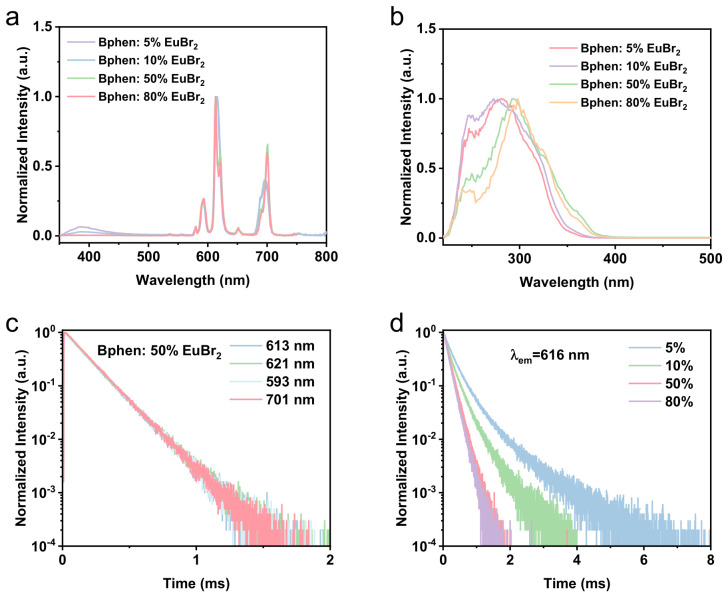
(**a**) PL spectra (excited at 300 nm) and (**b**) PLE spectra of blended films with blending ratios of 5%, 10%, 50%, and 80%; (**c**) time-resolved PL decay curves for the film with a blending ratio of 50%, monitored at 616 nm; (**d**) time-resolved PL decay curves of blended films with different EuBr_2_ blending ratios, monitored at the emission wavelength of 616 nm.

The luminescence properties of films with different blending ratios were further investigated. Figure 2a shows the PL spectra of the films denoted as Bphen: *x*% EuBr_2_ (*x* = 5, 10, 50, 80). Films with different blending ratios all exhibited similar emission characteristics, whereas the emission intensity of the Bphen ligand gradually decreased with increasing EuBr_2_ blending ratio. However, a high blending ratio also led to a decrease in the photoluminescence quantum yield (PLQY) of the films, as shown in Table 1. This indicates that although a higher EuBr_2_ ratio promotes coordination between Bphen and EuBr_2_ and thereby suppresses ligand emission, an excessive amount of EuBr_2_ induces a concentration quenching effect, which in turn reduces the luminescence efficiency. In addition, the fine structure of the PL spectra of Eu(III) can be observed. Bands peaking at approximately 579, 592, 616, 652 and 690–700 nm are attributed to the ^5^D_0_ to ^7^F_0_, ^5^D_0_ to ^7^F_1_, ^5^D_0_ to ^7^F_2_, ^5^D_0_ to ^7^F_3_ and ^5^D_0_ to ^7^F_4_ transitions, respectively. The ^5^D_0_ to ^7^F_0_ transition is allowed only in C_n_, C_nv_ and C_s_ site symmetries; its observation at 579 nm in films of all compositions therefore indicates low-symmetry coordination. And Bphen is a sterically hindered aromatic ligand, which is unable to form regular coordination polyhedra with high symmetries such as C_3_ and C_3h_. Therefore, the coordination structure is inferred to be a low-symmetry lattice with C_n_, C_nv_ or C_s_ symmetry. The ^5^D_0_ to ^7^F_1_ transition is predominantly magnetic-dipole allowed and hence only weakly dependent on the local field. By contrast, the ^5^D_0_ to ^7^F_2_ transition is a hypersensitive electric-dipole transition whose intensity is strongly governed by the local symmetry of the Eu(III) site. Under the proposed low-symmetry coordination, the ^7^F multiplet is expected to split into 3 to 5 sublevels. As the blend ratio increases, the interaction between Eu(III) and the ligand field tends to be stronger, leading to further energy-level splitting and the appearance of fine structure near 616 nm. Concurrently, the splitting of the ^5^D_0_ to ^7^F_4_ band in the 690–700 nm region becomes more pronounced [34]. The photoluminescence excitation (PLE) spectra of the films with different doping ratios are shown in Figure 2b. Increasing the EuBr_2_ ratio leads to a red shift in the excitation edge. This is because at low doping ratios, Bphen molecules greatly outnumber EuBr_2_ molecules; excitation light is mainly absorbed by Bphen and then transferred to the Bphen-EuBr_2_ complex via energy transfer to generate luminescence. At high blending ratios, the Bphen-EuBr_2_ complex becomes more abundant and directly absorbs the excitation light.

To further investigate the effect of blending ratio on the excited-state dynamics of the films, we measured the time-resolved photoluminescence (TRPL) decay curves of the films with different blending ratios. Taking the film with a blending ratio of 50% as an example, Figure 2c shows the TRPL decay curves monitored at emission wavelengths of 593, 613, 621 and 701 nm. The excited-state lifetimes at different emission wavelengths are identical, indicating that these distinct emission peaks all originate from the same principal energy level transition (from the ^5^D_0_ to the ^7^F_J_). The TRPL decay of films with different blending ratios was analysed at the emission wavelength of 616 nm, as shown in Figure 2d. As the EuBr_2_ ratio increases, the excited-state lifetime of the blending film shortens. The increased EuBr_2_ ratio reduces the distance between Eu(III) ions, promoting quenching among their long-lived excited states. This quenching also lowers the PLQY, consistent with Table 1, where higher blending ratios correspond to lower PLQY values.

**Figure 3:**
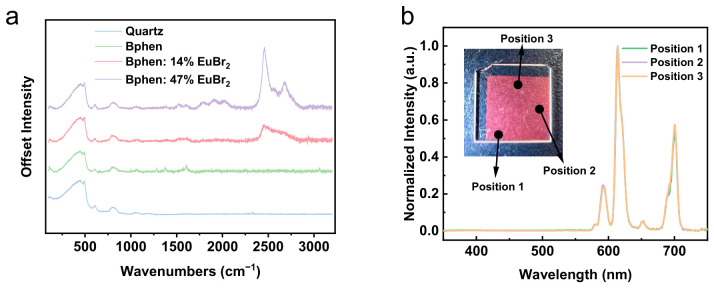
(**a**) Raman spectra of the quartz substrate, neat Bphen, and Bphen:EuBr_2_ films with EuBr_2_ ratios of 14% and 47%; (**b**) PL spectra measured at different positions on the film with a blending ratio of 47%.

Raman spectroscopy was used to characterize the structure of the blended films. Figure 3a shows the Raman spectra of the quartz substrate, neat Bphen, and Bphen:EuBr_2_ films with EuBr_2_ ratios of 14% and 47%. The film with 14% EuBr_2_ exhibits a peak at 2460 cm^−1^, while the film with 47% EuBr_2_ shows peaks at both 2460 cm^−1^ and 2679 cm^−1^. This indicates an interaction between Bphen and EuBr_2_ that strengthens with increasing blending ratio. The splitting of the emission peak into 613 nm and 622 nm at high blending ratios is attributed to this strong interaction. These results confirm that the luminescence of the blended films originates from the reaction between Bphen and EuBr_2_, and that uniform films are formed after the reaction. As shown in Figure 3b, the PL spectra excited at different positions on the film are nearly identical.

**Figure 4:**
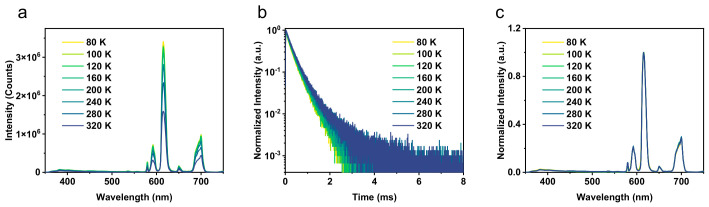
(**a**) PL spectra excited at 300 nm, measured at various temperatures; (**b**) Time-resolved PL decay curves monitored at 616 nm at different temperatures; (**c**) normalized PL spectra excited at 300 nm, recorded at various temperatures.

The temperature-dependent luminescence properties of the Bphen and EuBr_2_ blended films were further investigated. Figure 4a shows the PL spectra of the Bphen: 10% EuBr_2_ film at different temperatures. The emission intensity gradually increased as the temperature decreased. For the characteristic emission peak at 616 nm, the intensity at 80 K was 2.13 times that at 320 K. This phenomenon can be attributed to the effective suppression of phonon vibrations at low temperatures, which reduces the probability of nonradiative transitions and enhances the radiative transition rate. Correspondingly, Figure 4b shows that the excited state lifetime of the 616 nm emission was shorter at low temperature than at 320 K. The tail component of the decay curve further decreased at low temperature, and the decay curve exhibited an almost single exponential behaviour, confirming the suppression of phonon vibration processes at low temperature. Notably, after normalising the PL spectra collected at different temperatures, the spectral curves were found to be almost identical, as shown in Figure 4c. This indicates that the emission spectrum of the Bphen and EuBr_2_ blended film is remarkably temperature insensitive; that is, both the emission peak positions and the spectral curves remain stable over a wide temperature range.

**Figure 5:**
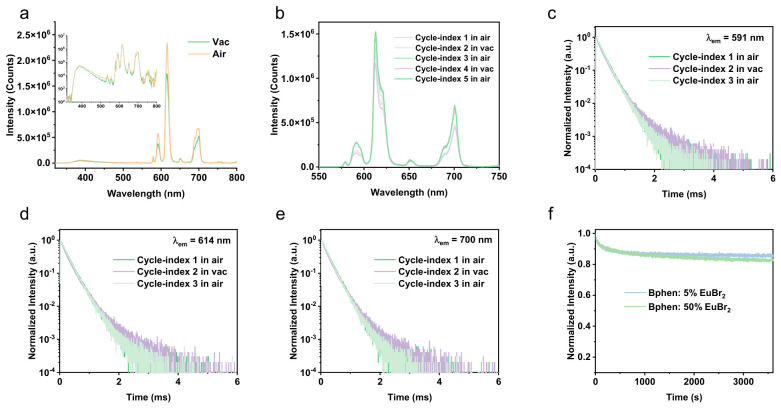
(**a**) PL spectra measured under 300 nm excitation for the blended film with a blending ratio of 10%, recorded in air and under vacuum; (**b**) PL spectra of the same film subjected to multiple test cycles; Time-resolved PL decay curves monitored at (**c**) 591 nm, (**d**) 614 nm, and (**e**) 700 nm, measured in air and under vacuum; (**f**) PL intensity decay curves monitored at 616 nm under 300 nm excitation for the composite films with blending ratios of 5% and 50%.

Figure 5a shows the PL spectra of the Bphen: 10% EuBr_2_ film measured in vacuum and in air. For the characteristic emission at 616 nm, the luminescence intensity of the film in air was approximately 1.36 times that in vacuum. The results of multiple cycle tests are shown in Figure 5b. The PL intensity in air was consistently higher than that in vacuum, and the luminescence response exhibited reversible behaviour, suggesting that the observed emission enhancement may originate from interactions between certain components in air and the blended film. TRPL decay curves were monitored at emission wavelengths of 591, 614 and 700 nm, as shown in Figure 5c–e. We found that the excited-state lifetime in air was shorter than that in vacuum, and the tail component of the decay curve was significantly reduced. Based on the above observations, we propose that oxygen molecules may increase the efficiency of energy transfer from the organic ligand to the Eu(III) ions in the blended film, thereby promoting the radiative transition process and shortening the excited-state lifetime.

To further investigate the stability of the blended films under continuous photoexcitation, we monitored the change in luminescence intensity at 616 nm under continuous excitation at 300 nm, as shown in Figure 5f. For films with low (5%) and high (50%) blending ratios, the PL intensity decreased rapidly at first, then the rate of decrease slowed markedly under prolonged excitation. After 10 min of continuous excitation, the PL intensities of the films with 5% and 50% blending ratios remained at 0.8857 and 0.8786 of their initial values, corresponding to decreases of 11.43% and 12.14%, respectively. During the subsequent excitation from 10 to 60 min, the PL intensities further decreased to 0.8552 and 0.8230 of the initial values, with corresponding decreases of 3.44% and 6.33%, respectively. These results indicate that the as-prepared blended films exhibit reasonable stability, and that the film with a lower blending ratio shows relatively better stability than that with a higher blending ratio.

**Figure 6:**
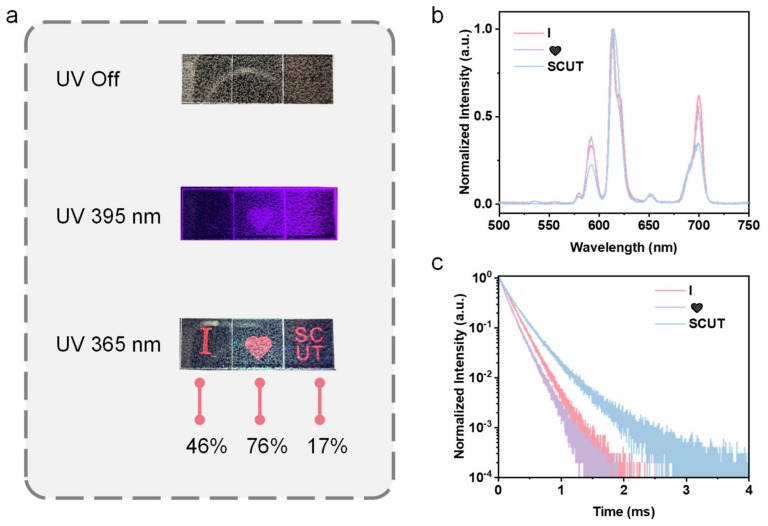
(**a**) Photographs of luminescence from patterns with different shapes under room light, 395 nm, and 365 nm handheld UV lamps; No characteristic luminescence emission was detected from the films under 395 nm UV irradiation; the visible purple hue in the photograph arises from the reflected light of the excitation lamp source.; (**b**) photoluminescence spectra of the patterned films excited at 300 nm; (**c**) time-resolved photoluminescence decay curves monitored at 613 nm for the different patterns.

Given the consistency of the PL spectra of the blended films at different temperatures and their good photostability in air, we further investigated their application in anti-counterfeiting. Patterned blended films with different blending ratios were prepared, as shown in Figure 6a. Three patterned films with EuBr_2_ mixing ratios of 46%, 76% and 17% were directly fabricated on a quartz substrate, designed as “I”, “♥”, and “SCUT”, respectively. Under ordinary fluorescent lamp illumination, almost only the quartz substrate was visible, whereas the thin film was barely visible. No luminescence was observed from the films under a 395 nm UV lamp, but under 365 nm UV excitation, the films exhibited distinct red emission. The PL emission spectra of the patterned films under 300 nm excitation are shown in Figure 6b. The ratio of the emission intensity at 613 nm to that at 621 nm was extracted as an identifying feature, as listed in Table 2. This ratio increased with increasing EuBr_2_ ratio, indicating that each mixing ratio corresponds to a unique intensity ratio. This allows the establishment of a database linking the blending ratio used in fabrication to the intensity ratio for anti-counterfeiting purposes. Furthermore, the excited state lifetimes of the films with different blending ratios are shown in Figure 6c and Table 2. For the “I” pattern, the excited state lifetime was 178 μs; for the “♥” pattern, 150 μs; and for the “SCUT” pattern, 256 μs. The excited state lifetime decreased with increasing blending ratio. Thus, both the emission intensity ratio and the excited-state lifetime can be used to verify the evaporation fabrication process, enabling dual authentication anti-counterfeiting.

## 4. Conclusions

In summary, we have developed a direct “deposition-as-synthesis” approach using GPVEA to fabricate organic–inorganic europium-based hybrid nanomaterials, using divalent EuBr_2_ and Bphen as the precursors. Notably, the as-prepared films exhibit characteristic sharp emission peaks originating from Eu(III), indicating an in situ oxidation from Eu(II) to Eu(III) during the co-deposition process. By tuning the blending ratio of Bphen to EuBr_2_, the emission properties and excited state dynamics can be modulated, with the optimal PLQY (8.27%) observed at a 10% EuBr_2_ ratio. The blend films show remarkable spectral stability over a wide temperature range (80 K to 320 K) and exhibit higher luminescence intensity in air than in vacuum. Moreover, the films demonstrate good photostability, with only 3.44% decay over 50 min under 300 nm excitation. Leveraging these advantages, patterned films with different blending ratios were fabricated, enabling dual-mode anti-counterfeiting based on both emission intensity ratio and excited-state lifetime. This work provides a facile and vacuum-compatible route for preparing europium-based luminescent nanomaterials via GPVEA, opening new opportunities for optical anti-counterfeiting and potential optoelectronic applications.

## Figures and Tables

**Table 1:** PLQY at different blending ratios.

**Ratio**	5%	10%	30%
**PLQY ^1^**	1.74%	8.27%	5.62%

^1^ Integration from 500 nm to 750 nm.

**Table 2:** Intensity ratios and excited state lifetimes for the patterns with different shapes.

Pattern	I	♥	SCUT
**Intensity ratio ^1^ [a.u.]**	1.61	1.93	1.53
**Lifetime ^2^ [μs]**	178	150	256

^1^ The intensity ratio is defined as the ratio of intensities at 613 nm and 621 nm; ^2^ Average lifetime of the excited-state monitored at 613 nm.

## Data Availability

All data supporting the findings of this study are provided within the article.

## Abbreviations

The following abbreviations are used in this manuscript:

GPVEA	Gas-phase vacuum evaporation approach
ALD	Atomic layer deposition
OLEDs	Organic light-emitting diodes
PL	Photoluminescence
PLQY	Photoluminescence quantum yield
PLE	Photoluminescence excitation
TRPL	Time-resolved photoluminescence
